# Antibacterial Effects and Biocompatibility of Titania Nanotubes with Octenidine Dihydrochloride/Poly(lactic-co-glycolic acid)

**DOI:** 10.1155/2015/836939

**Published:** 2015-05-19

**Authors:** Zhiqiang Xu, Yingzhen Lai, Dong Wu, Wenxiu Huang, Sijia Huang, Lin Zhou, Jiang Chen

**Affiliations:** ^1^School of Stomatology, Fujian Medical University, Fuzhou, Fujian 350000, China; ^2^Department of Oral Medicine, Xiamen Medical College, Xiamen, Fujian 361000, China; ^3^Department of Oral Implantology, Affiliated Stomatological Hospital of Fujian Medical University, Fuzhou, Fujian 350002, China

## Abstract

Titanium (Ti) implants with long-term antibacterial ability and good biocompatibility are highly desirable materials that can be used to prevent implant-associated infections. In this study, titania nanotubes (TNTs) were synthesized on Ti surfaces through electrochemical anodization. Octenidine dihydrochloride (OCT)/poly(lactic-co-glycolic acid) (PLGA) was infiltrated into TNTs using a simple solvent-casting technique. OCT/PLGA-TNTs demonstrated sustained drug release and maintained the characteristic hollow structures of TNTs. TNTs (200 nm in diameter) alone exhibited slight antibacterial effect and good osteogenic activity but also evidently impaired adhesion and proliferation of bone marrow mesenchymal stem cells (BMSCs). OCT/PLGA-TNTs (100 nm in diameter) supported BMSC adhesion and proliferation and showed good osteogenesis-inducing ability. OCT/PLGA-TNTs also exhibited good long-term antibacterial ability within the observation period of 7 d. The synthesized drug carrier with relatively long-term antibacterial ability and enhanced excellent biocompatibility demonstrated significant potential in bone implant applications.

## 1. Introduction

Titanium (Ti) implants are widely used clinically because of their high biocompatibility and good mechanical properties [1]. However, implant-associated infections remain as one of the most serious postoperative complications [2]. The high incidence of implant-associated infections can be mainly attributed to the adhered bacteria that form a biofilm; this biofilm provides the bacteria with high resistance to host defenses and antimicrobial therapies [3, 4]. Conventional systemic drug therapies in bones present limitations such as low efficacy, poor bioavailability, and toxicity [5]. Thus, localized delivery of antimicrobial agents with time-effective handling of infection while potentially eliminating problems associated with systemic administration is highly desirable [6, 7]. Furthermore, Ti bioactivity is not ideal and thus may lead to the formation of a fibrous capsule around the implant [5]. Fibrous tissues can prevent the contact between host immunity sentinel cells and bacteria [2]. Hence, implant coatings with enhanced osteogenic activity and antibacterial property must be used to prevent infections and elongate the service life of Ti implants.

With the advent of nanotechnology, nanostructured materials play fundamental roles in orthopedic research because bones demonstrate a structural hierarchy at the first level in the nanometer regime [8]. Titania nanotubes (TNTs) fabricated on the Ti surface through electrochemical anodization have received considerable attentions in orthopedic research, because of their high bioactivity to promote bone cell growth and cell differentiation, unlike unanodized Ti [9–12]. Moreover, TNTs with controllable dimensions, high surface-to-volume ratio, and hollow structures have been demonstrated to be superior platforms for local antibiotic delivery applications [13–17].

TNTs, as antibiotic carriers, are challenged with two disadvantages that must be addressed before clinical applications. First, the drug release in proposed drug-delivery systems is directed through diffusion of drug molecules from the nanotube structure [5]. The release rate is very fast and short, thus limiting the antimicrobial effects to early-stage peri-implant infections. Although polymers and phospholipids were directly coated on the top of drug-loaded TNTs to extend drug release, TNTs were buried in the coating cap; hence, the potential benefit of TNTs in promoting bone growth was diminished [18–20]. Poly(lactic-co-glycolic acid) (PLGA) has been loaded into TNTs through solvent-casting technique to maintain their characteristic hollow structure and sustain drug release [21]. However, to the best of our knowledge, the biocompatibility of TNTs loaded with PLGA remains unknown. Second, bacteria are highly adaptable in nature, which leads to the evolution of strains resistant to conventional antibiotics [22]. Agents working through unspecific modes of action are required to overcome this resistance such as octenidine dihydrochloride (OCT). OCT, approved as a medicinal substance in several European countries, is an established bispyridine antiseptic with a broad activity and is commonly used as wound antiseptic [23, 24]. It has attracted increasing attentions because of broad antibacterial spectrum including antibiotic-resistant bacteria, noncytotoxicity at suitable doses, satisfactory stability, and smaller possibility to develop resistant bacteria [25, 26].

In this study, we investigated the possibility of using composite PLGA-TNTs as a carrier for sustained OCT delivery. Despite active studies on using localized delivery of antimicrobial agents to prevent implant infections, there have been few reports on using antiseptics [27] and none on using nanotubes to deliver antiseptics. In this paper, for the first time, OCT/PLGA was loaded into TNTS through a simple dip-coating process. Sustained antibacterial ability and biocompatibility were systematically investigated.

## 2. Materials and Methods

### 2.1. Fabrication of TNTs on Ti

TNTs were fabricated through anodization on a Ti sheet. Ti samples (Alfa-Aesar, Ward Hill, MA, USA; 1 × 1 × 0.025 cm^3^, 99.8% purity) were degreased by sonication in acetone and deionized water for 15 min. The samples were then eroded for 10 s in 4 wt% HF–5 mol/L HNO_3_, followed by rinsing with deionized water and drying in air. A two-electrode electrochemical cell using a platinum sheet as the counter-electrode was used. Anodization was performed using a mixture of 0.50 wt% NH_4_F + 10 vol% H_2_O in glycerol at 60 V for 5 h. After anodization, the samples were rinsed with deionized water, dried in air, and annealed at 450°C for 2 h.

### 2.2. OCT Loading

Two types of samples were prepared. The first type was prepared by loading the mixture of OCT/PLGA into TNTs. PLGA (Sigma-Aldrich, St Louis, MO, USA; lactide-to-glycolide ratio: 65 : 35, 24,000 Da–38,000 Da) and OCT (TCI, Shanghai, China) were dissolved in dichloromethane at 15 mg/mL and 0.5 *μ*g/mL, respectively. The polymer/drug mixture was loaded into TNTs using a dip-coating process at 40°C for 2 d and then dried in air. The second type was produced by directly loading OCT into TNTs. OCT was dissolved in low-surface tension solvent (ethanol) and forced into TNTs using a vacuum-assisted physical adsorption method. In brief, 50 *μ*L of 40 *μ*g/mL OCT solution was pipetted onto the nanotube surfaces and allowed to dry under a vacuum desiccator at 20°C for 1 h. The loading process was repeated three times.

### 2.3. Specimen Characterization

Scanning electron microscopy (SEM) (JSM-7500F; JEOL, Tokyo, Japan) was used to characterize the morphology of the prepared TNTs and OCT/PLGA-TNTs. The infiltration of PLGA polymer into TNTs was also assessed by SEM according to the report of Jia and kerr [21]. Scotch tape was used to separate the OCT/PLGA-TNTs from Ti foil. The tape with OCT/PLGA-TNTs then was soaked in 5 vol% HF solution for 15 min until all TNTs were etched off. The remaining PLGA was examined through SEM.

### 2.4. Contact Angle Determination

A 1 *μ*L drop of distilled water was delivered on the clean specimen surface with a syringe at a room temperature and in the open atmosphere of the lab. Contact angles were measured on the obtained photographs (Phoenix 300; SEO, Seoul, Korea). The mean value was calculated from five separate measurements.

### 2.5. Protein Adsorption Assay

A 1 mL droplet of DMEM-LG containing 10% FCS was pipetted onto each specimen. After incubation at 37°C for 2 h, the proteins adsorbed onto the samples were detached using 1% sodium dodecyl sulfate. The protein concentrations were determined using a MicroBCA protein assay kit (Pierce, Rockford, IL, USA).

### 2.6. Release Profile of OCT

OCT has been shown to be stable in chloroform at 40°C [25]. So, in vitro OCT release kinetics of the samples were measured using ultraviolet visible spectroscopy (UV-1750; Shimadzu, Kyoto, Japan) by recording the absorption peak at 280 nm, which is the characteristic excitation wavelength of OCT. Two specimens, TNTs loaded with OCT and TNTs loaded with OCT/PLGA, were immersed in 1-mL of PBS in a glass vial while rotating (60 revolutions per minute) at 37°C. After 1, 2, 4, and 6 h, as well as after 1, 2, 3, 6, 9, 12, 15, and 18 d, 500 mL of the solution was sampled and fresh PBS was replenished. Three samples were tested in each time interval, and the mean value was used in data analysis.

### 2.7. Bacteria Cultures

*Staphylococcus aureus* (*S. aureus*) (ATCC25923; American Type Culture Collection, Manassas, VA, USA) was cultivated in the brain-heart infusion broth medium at 37°C for 12 h and then adjusted to a concentration of 10^6^ CFU/mL. The specimens were placed on 24-well culture plates and separately incubated in 1 mL of the bacteria-containing medium.

#### 2.7.1. SEM Observation

After being incubated in 1 mL of the bacteria-containing medium for 6 h, the samples were rinsed with PBS, fixed with 3% glutaraldehyde, dehydrated in graded ethanol series, freeze-dried, sputter-coated with thin platinum layers, and observed by SEM.

#### 2.7.2. Antibacterial Assay

In vitro antibacterial activity was assessed by the plate-counting method. After culturing for 1, 4, and 7 days, the sample was rinsed in PBS and ultrasonically agitated to detach the bacteria from the sample. The bacteria suspensions were recultivated on agar plates for colony counting. The antibacterial rates were calculated using the following formula: antibacterial rate (%) = (CFU of control − CFU of experimental groups)/CFU of control × 100%, where Ti served as the control while TNTs and TNTs/PLGA constituted the experimental groups.

### 2.8. Cell Cultures

Sprague-Dawley rat bone marrow mesenchymal stem cells (BMSCs) were purchased from Cyagen Biosciences (Guangzhou, China). The cells were cultured in DMEM-LG containing 10% FCS at 37°C, and the medium was changed every 3 d. Cells were used between passage 4 and passage 6 in the following experiments. The samples were placed in 24-well plates, and the BMSCs were seeded at a density of 4 × 10^4^/well for the cell adhesion assay and 2 × 10^4^/well for the other assays.

#### 2.8.1. Cell Morphology

After culturing for 2 d, the samples were rinsed with PBS, fixed with 2.5% glutaraldehyde, dehydrated in graded ethanol series, freeze-dried, sputter-coated with thin platinum layers, and observed by SEM.

#### 2.8.2. Adhesion and Proliferation

For the cell adhesion assay, the adherent cells were fixed and stained with 4,6-diamidino-2-phenylindole (DAPI; Sigma-Aldrich) after culturing for 0.5, 1, and 2 h. Images were captured from five random fields by a fluorescence microscope, and the cell number in each field was determined. To assess cell proliferation, the cell numbers were assessed using Cell Counting Kit-8 (CCK-8; Beyotime, Shanghai, China) assay after seeding for 1, 3, and 7 d.

#### 2.8.3. Gene Expressions

The expression levels of osteogenesis-related genes, including runt-related transcription factor 2 (RUNX2, a key transcript factor for osteogenic differentiation), alkaline phosphatase (ALP, an early marker for osteogenic differentiation), osteocalcin (OCN, a late marker for osteogenic differentiation), and type 1 collagen (COL-1, a main collagen found in bones), were measured using quantitative reverse transcription polymerase chain reaction (qRT-PCR). Total RNA was extracted using Trizol (Invitrogen, Carlsbad, CA, USA) after culturing for 2 weeks. Total RNA was then reverse-transcribed with a cDNA Reverse Transcription Kit (TaKaRa, Shiga, Japan), and qRT-PCR analysis was performed on an ABI Prism 7500 real-time PCR cycler (Applied Biosystems, Carlsbad, CA, USA) using SYBR Premix Ex Taq II (TaKaRa). The primers for the target genes are listed in Table 1. The expression levels of the target genes were normalized to that of the housekeeping gene GAPDH.

### 2.9. Statistical Analysis

All data were expressed as the mean ± standard deviation (SD). One-way ANOVA and Student-Newman-Keuls post hoc test were used to determine the level of significance. *P* < 0.05 was considered to be significant, and *P* < 0.01 was considered to be highly significant.

## 3. Results

### 3.1. Specimen Characterization

**Figure 1:**
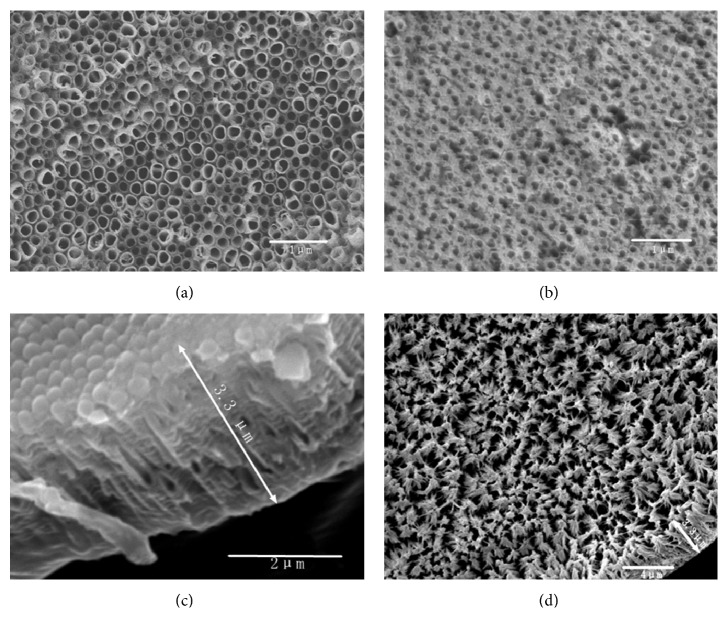
SEM images: (a) TNTs with a diameter of 200 nm, (b) OCT/PLGA-TNTs with a diameter of 100 nm, (c) side view of TNTs, and (d) remaining PLGA.

The SEM images of TNTs with and without OCT/PLGA loading are shown in Figure 1. TNTs (200 nm in diameter) were neatly and uniformly arranged over the anodized Ti surface in Figure 1(a). The diameter decreased to 100 nm after OCT/PLGA loading, and the intertubular areas were filled with PLGA, as shown in Figure 1(b). The side view of TNTs and the image of the remaining PLGA after TNTs removal are shown in Figures 1(c) and 1(d), respectively. The remaining PLGA was uniformly arranged in most of the areas, and its length was similar to the length of TNTs. Thus, PLGA demonstrated an excellent infiltration depth into TNTs.

### 3.2. Static Contact Angles

**Figure 2:**
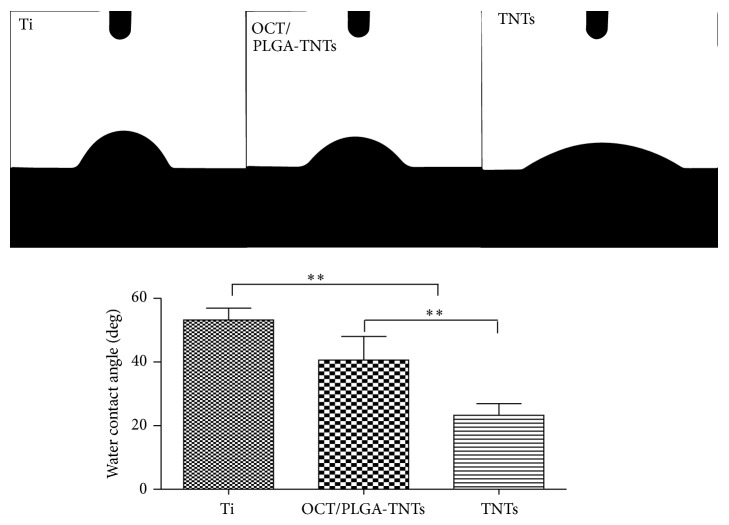
Photographs of water droplets on different samples and multiple-comparison results of contact angles on different samples (mean ± SD, *N* = 5, ^*^ *P* < 0.05, ^**^ *P* < 0.01).

Drop images were captured by a video camera in the direction perpendicular to the surface (Figure 2). For hydrophilic material, the contact angle is lower than 90 degrees, and the smaller the contact angle, the greater the hydrophilicity. Multiple comparisons (Figure 2) revealed that TNTs showed the greatest surface hydrophilicity, whereas Ti exhibited the greatest surface hydrophobicity.

### 3.3. Protein Adsorption Assay

**Figure 3:**
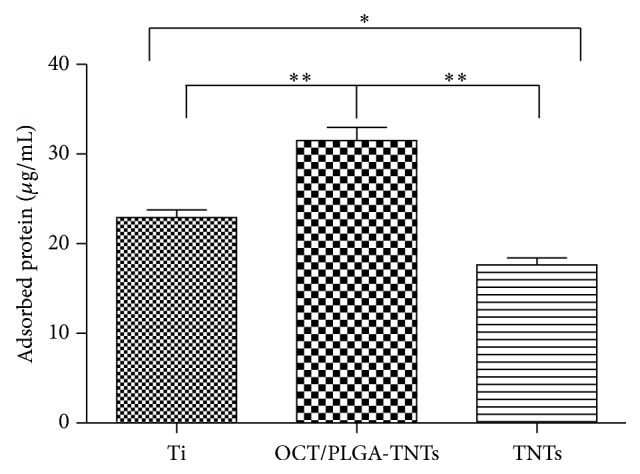
Protein adsorption to the samples after 2 h of immersion in DMEM-LG containing 10% FCS (mean ± SD, *N* = 3, ^*^ *P* < 0.05, ^**^ *P* < 0.01).

The amounts of adsorbed proteins from 10% FCS after 2 h of incubation are presented in Figure 3 to elucidate subsequent cellular responses. OCT/PLGA-TNTs with higher hydrophilicity absorbed more proteins than control Ti. However, paradoxically, TNTs with the highest hydrophilicity had the fewest protein aggregates. It is because the protein aggregates (≈30-nm-size regime) initially attach only to the available surfaces that are the top portion of the nanotube walls [11, 12], and these aggregates are too small to anchor on TNTs with a diameter of 200 nm. The protein aggregates could easily attach to OCT/PLGA-TNTs, probably because the larger intertubular areas, which were filled with PLGA, provided more effective nucleation sites for protein adsorption.

### 3.4. OCT Release

**Figure 4:**
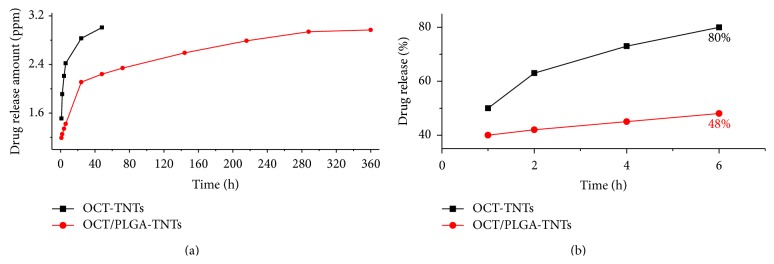
In vitro release profiles in PBS: (a) accumulative amount of OCT release from the samples and (b) accumulative percentage of OCT release from the samples.

Figure 4 shows the OCT release time profiles from TNTs and OCT/PLGA-TNTs in PBS. For the OCT directly released from the nanotubes, the release kinetics could be described in two phases: a high percentage (80%) of the drug released in the first 6 h (initial burst release) and then a slow release for the following 2 d. By contrast, in the case of PLGA-TNTs, the drug release pattern was directed through the transport of drug through the polymer matrix and the rate of polymer degradation [19, 21], so the burst release decreased from 80% to 48% (versus TNTs) and the extended overall release increased from 2 d to 15 d.

### 3.5. Antimicrobial Activity

**Figure 5:**
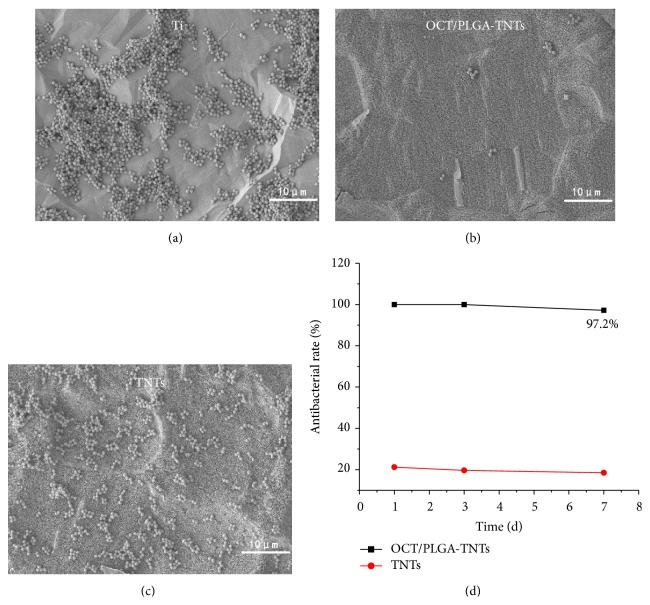
SEM images of* S. aureus* incubated for 6 h on the samples: (a) control Ti, (b) OCT/PLGA-TNTs, and (c) TNTs. (d) shows in vitro antibacterial rate profiles of the samples.

Figures 5(a), 5(b), and 5(c) show the qualitative SEM assessments of bacteria incubated with samples. It could be observed that many multiple bacterial colonies formed colony masses on the surfaces of Ti and TNTs. In strong contrast, very few single bacterial colonies were detected on OCT/PLGA-TNTS.

Antibacterial activity was evaluated for 7 d as shown in Figure 5. On day 1, OCT/PLGA-TNTs loaded with OCT generated a high antibacterial rate of 100%. Although a slight decrease in the antibacterial rates was observed as time increased, a high antibacterial rate of 97.2% was maintained until day 7. This finding suggests an effective and long-term antibacterial activity against* S. aureus*. TNTs alone exhibited slight antibacterial rate of about 20%, which was relatively constant with time.

### 3.6. Cell Morphology

**Figure 6:**
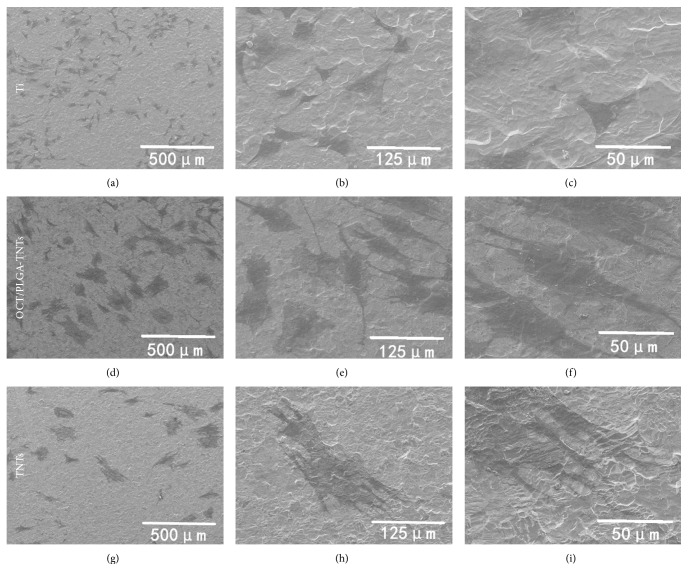
SEM pictures with three different magnifications of 200x (the first column), 800x (the second column), and 2,000x (the third column) showing the morphology of BMSCs after 2 d of culture on samples.

The BMSCs displayed dramatically different shapes related to the topography of the substrate, as shown in Figure 6. Most of the BMSCs on Ti appeared round and spread poorly with no cellular extensions and filopodia propagation indicative of undifferentiated BMSCs (Figures 6(a), 6(b), and 6(c)). By contrast, the cells on OCT/PLGA-TNTs (Figures 6(d), 6(e), and 6(f)) and TNTs (Figures 6(g), 6(h), and 6(i)) had to extend across the tubes to locate a protein-deposited surface on intertubular areas for initial contact, thereby becoming more extended with a large number of prominent filopodia and unidirectional lamellipodia extensions compared with Ti, and pure TNTs with a larger diameter presented stronger induction.

### 3.7. Adhesion and Proliferation

**Figure 7:**
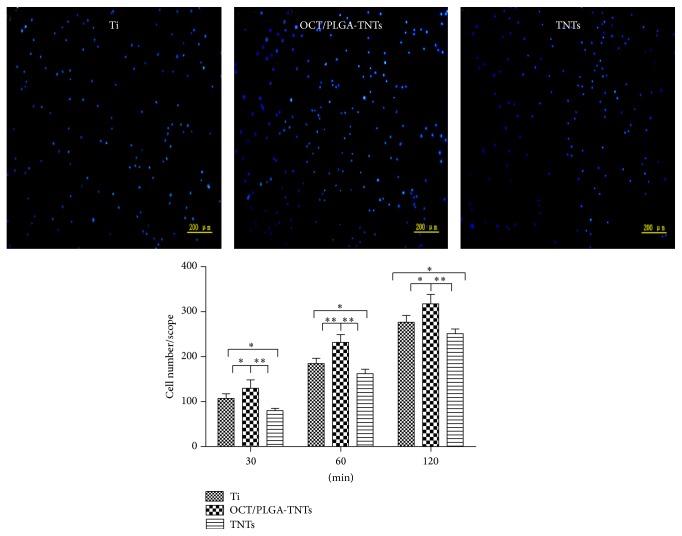
Fluorescence images of initial adherent BMSCs stained with DAPI after 1 h and cell numbers measured by counting cells for 0.5, 1, and 2 h (mean ± SD, *N* = 5, ^*^ *P* < 0.05, ^**^ *P* < 0.01).

The highest numbers of initial adherent cells on OCT/PLGA-TNTs and lowest numbers of initial adherent cells on TNTs are shown in Figure 7. Upon contact of an implant surface with blood, the proteins available in the serum adsorb to the surface within an initial incubation time and mediate the subsequent cellular performance [11, 28]. Cell attachment is believed to be significantly greater on material surfaces with more protein adhesions [29–33]. Thus, OCT/PLGA-TNTs with more protein adhesions induced more cell attachment at an early time than Ti, and TNTs with the fewest protein aggregates induced the least attachment.

**Figure 8:**
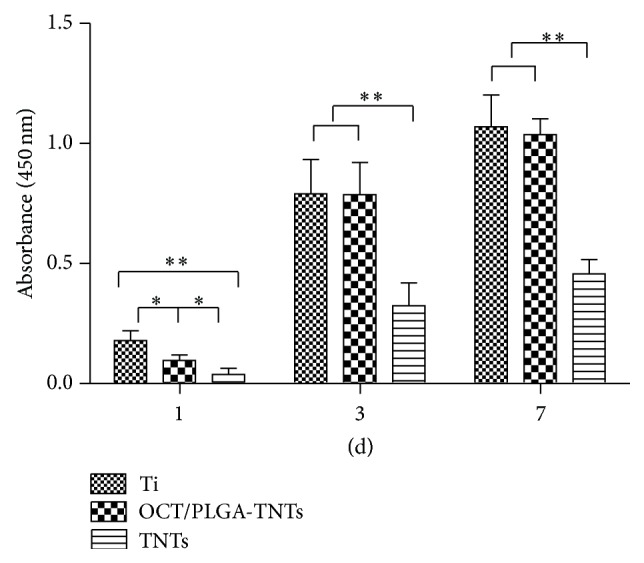
Cell proliferation measured by CCK-8 assay after culturing BMSCs on three different samples for 1, 3, and 7 d (mean ± SD, *N* = 3, ^*^ *P* < 0.05, ^**^ *P* < 0.01).

Cell proliferation was measured by the CCK-8 assay (Figure 8). On day 1, the cell numbers on OCT/PLGA-TNTs were slightly smaller than those on Ti. This slight proliferation suppressive effect was possibly related to the differentiation tendency of BMSCs because of the reciprocal relationship between cell proliferation and differentiation [34, 35]. The inhibition of BMSC proliferation was not serious or long. By days 3 and 7, the cell growth on 100 nm OCT/PLGA-TNTs caught up because of the large surface area available for cell colonization. This finding indicates that OCT/PLGA-TNTs did not impair cell viability and could support cell proliferation. However, cell proliferation on TNTs was obviously lower than that on the Ti control and OCT/PLGA-TNTs, and this trend became more evident with time. The very low adhesion at the early stage can potentially lead to cell quiescence or even apoptosis by anoikis, a type of programmed cell death through “homelessness” [36].

### 3.8. Gene Expressions

**Figure 9:**
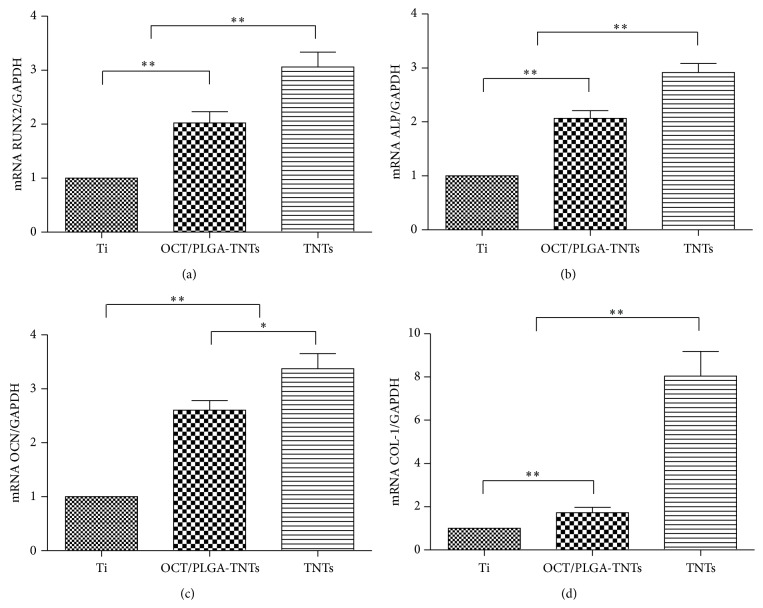
Relative expressions of (a) RUNX2, (b) ALP, (c) OCN, and (d) COL-1 by BMSCs seeding on different substrates for 2 weeks (mean ± SD, *N* = 3, ^*^ *P* < 0.05, ^**^ *P* < 0.01).

The expression levels of osteogenesis-related genes including RUNX2, ALP, OCN, and COL-1 were assessed by qRT-PCR. The results are shown in Figure 9. The three topographies explored in this study induced different gene expression levels. Previous study reported that elongated BMSCs are prone to undergo osteogenesis [37, 38], so the effects of nanotubes on inducing fast and good distribution of BMSCs favored the osteogenic ability. OCT/PLGA-TNTs and TNTs significantly promoted the expression of osteogenesis-related genes and demonstrated excellent osteogenic activity, with the latter exhibiting a higher promotion.

## 4. Discussions

With the steadily increasing demand for implants, a proper approach that can endow biomaterials with long-term antibacterial ability and biointegration has been actively pursued [28]. In the present study, OCT/PLGA was loaded into TNTs using a simple solvent-casting technique. The design sustained OCT release and maintained the characteristic hollow structure of TNTs. OCT/PLGA-TNTs showed good antibacterial characteristics and biointegration and are thus important to prevent implant-associated infections.

PLGA, which was approved by the US FDA as therapeutic material, was selected as the OCT carrier in this study because of its excellent biocompatibility and biodegradability without interrupting osseointegration [39]. As expected, our proposed design enabled slow and sustained release for 15 d using the composite OCT/PLGA-TNTs.* S. aureus*, known for its extensive resistance to antibiotics, is the most common cause of implant infections [40] and was therefore chosen for the study. The antibacterial ability of the OCT/PLGA-TNTs decreased gradually with time and the tendency was consistent with the OCT time release profiles. A postimplantation period of 6 h has been identified as the “decisive period” when the implant is particularly susceptible to surface colonization [28]. The very few single bacterial colonies in Figure 5(b) (versus many multiple bacterial colonies in Figures 5(a) and 5(c)) showed that the burst OCT release of 48% in the first 6 h could effectively inhibit bacterial adhesion on the OCT/PLGA-TNT samples, though the larger OCT release of 80% of TNTs may have resulted in less bacteria adhesion on the TNTs. After this stage, few drugs are needed to prevent further infection with the help of the host defense [41]. The antibacterial assay showed that the slow and sustained OCT release from OCT/PLGA-TNTS also exhibited a good long-term antibacterial ability within the observation period of 7 d. The long-term antibacterial ability with slow and sustained OCT release is meaningful to preventing implant infection while avoiding potential side effects associated with OCT overdose in clinical practice. Our results also showed that TNTs without drug loading slightly inhibited the antibacterial activity. This finding is in accordance with reports that controlled titania nanotube formation through anodization and heat treatment, which decrease the contact angles of water and form crystalline TiO_2_, leading to a decreased bacterial adhesion on them [42].

BMSCs are multipotent stem cells that can give rise to various adult cell types, including osteoblasts [43], and most osteoblastic cells that colonize the implant surface to induce bone growth originate from BMSCs [44]. Hence, it is crucial to evaluate the behavior of BMSCs on OCT/PLGA-TNTs before the clinical application. Cell assay showed that OCT/PLGA-TNTs could support BMSCs adhesion and proliferation. This finding indicates that excellent antibacterial ability can be obtained from OCT at concentrations lower than the values to induce BMSC damage. This result is in accordance with previous reports [25, 45], in which OCT is more toxic to* S. aureus* than to mammalian cells under similar test conditions, resulting in a good biocompatibility with concomitant antibacterial activity. Furthermore, OCT/PLGA-TNTs promoted cell spreading and thus demonstrated excellent osteogenic activity. Although PLGA can be replaced by serum proteins and extracellular matrices after degradation, the initial changes (within the first 24 h) in the cell shape and cytoskeletal distribution would decide the fate of stem cell differentiation [46]. In addition, a good cell distribution on the biomaterial surface is important in winning the “race for the surface” against bacteria, thereby aiding to combat infection [47]. Although TNTs alone showed the best ability to promote cell spreading and osteogenic differentiation, it obviously impaired cell adhesion and proliferation.

The technique reported in this study can be readily extended to other types of local drug-delivery applications to produce desirable biological effects. The incorporated amount and release rates can also be readily controlled by varying the drug concentration and the lactide-to-glycolide ratio.

## 5. Conclusions

In summary, OCT/PLGA was loaded into TNTs without completely filling the nanotubes using a simple solvent-casting technique to obtain sustained OCT release. OCT/PLGA-TNTs showed good antibacterial effects which could prevent postoperation complications and even late cases of infection while osteogenic activity was enhanced. Moreover, the fabrication process of OCT/PLGA-TNTs is simple, economical, and versatile. Thus, OCT/PLGA-TNTs are highly attractive for biomedical implants because of their controlled drug release, long-term antibacterial efficacy, and promotion of osseointegration.

## Supplementary Material

Graphical abstract showing the synthetic processes and properties of OCT/PLGA-TNTs.

## Figures and Tables

**Table 1:** Primers used for qRT-PCR.

Gene	Forward primer sequence (5′-3′)	Reverse primer sequence (5′-3′)
RUNX2	CCTCTGACTTCTGCCTCTGG	GATGAAATGCCTGGGAACTG
ALP	GCCTGGACCTCATCAGCATT	AGGGAAGGGTCAGTCAGGTT
OCN	CAAGTCCCACACAGCAACTC	CCAGGTCAGAGAGGCAGAAT
COL-I	ATCTCCTGGTGCTGATGGAC	GCCTCTTTCTCCTCTCTGACC
GAPDH	GGCACAGTCAAGGCTGAGAATG	ATGGTGGTGAAGACGCCAGTA
