# Supplementary figures and images for: Antibacterial Effects and Biocompatibility of Titania Nanotubes with Octenidine Dihydrochloride/Poly(lactic-co-glycolic acid)
Source: Biomed Res Int. 2015 May 19;2015:836939. doi: 10.1155/2015/836939 (PMC4452295; doi:10.1155/2015/836939)

## Graphical abstract

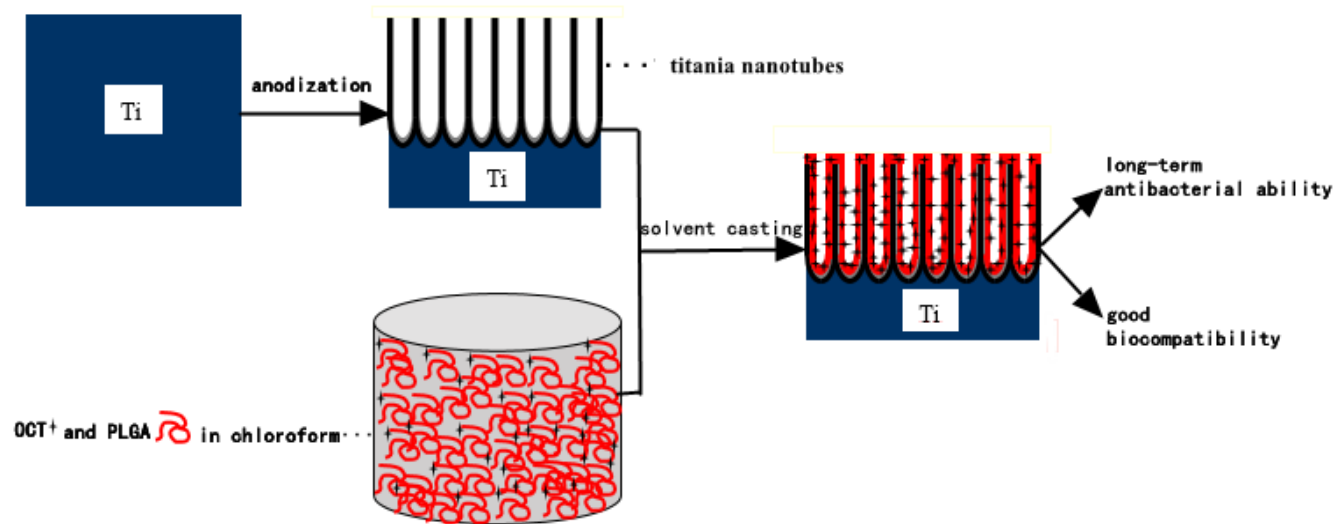

Supplement: Supplementary file 1 — Graphical abstract showing the synthetic processes and properties of OCT/PLGA-TNTs. [file 836939.f1.pdf]
